# Individual and community level factors associated with use of iodized salt in sub-Saharan Africa: A multilevel analysis of demographic health surveys

**DOI:** 10.1371/journal.pone.0251854

**Published:** 2021-05-17

**Authors:** Yigizie Yeshaw, Alemneh Mekuriaw Liyew, Achamyeleh Birhanu Teshale, Tesfa Sewunet Alamneh, Misganaw Gebrie Worku, Zemenu Tadesse Tessema, Adugnaw Zeleke Alem, Getayeneh Antehunegn Tesema

**Affiliations:** 1 Department of Physiology, School of Medicine, College of Medicine and Health Sciences, University of Gondar, Gondar, Ethiopia; 2 Department of Epidemiology and Biostatistics, Institute of Public Health, College of Medicine and Health Sciences, University of Gondar, Gondar, Ethiopia; 3 Department of Human Anatomy, School of Medicine, College of Medicine and Health Sciences, University of Gondar, Gondar, Ethiopia; Boston University School of Medicine, UNITED STATES

## Abstract

**Introduction:**

Iodine deficiency disorder a common problem in sub-Saharan Africa (SSA). It affects not only the health of the affected individual but also the economic development of the country. However, to the best of our knowledge, there is a scarcity in literature about the associated factors of iodized salt utilization in sub-Saharan Africa. Therefore, this study aimed to identify both individual and community level determinants of iodized salt utilization in sub-Saharan Africa.

**Methods:**

This study used the appended datasets of the most recent demographic and health survey from 31 sub-Saharan countries. A total weighted sample of 391,463 households was included in the study. Both bivariable and multivariable multilevel logistic regression were done to determine the associated factors of iodized salt utilization in SSA. P value ≤ 0.05 was used to declare statistically significant variables.

**Results:**

Those households with primary (AOR  =  1.53, 95% CI  =  1.50–1.57), secondary (AOR  = 1.81, 95% CI  =  1.76–1.86) and higher education level (AOR  =  2.28, 95% CI  =  2.17–2.40) had higher odds of iodized salt utilization. Households with middle (AOR  =  1.05, 95% CI  =  1.02–1.08), richer (AOR  = 1.13, 95% CI  =  1.09–1.17) and richest wealth index (AOR  =  1.23, 95% CI  =  1.18–1.28) also had an increased chance of using iodized salt. Households from high community media exposure (AOR  =  2.07, 95% CI  =  1.71–2.51), high community education level (AOR  =  3.78, 95% CI  =  3.14–4.56), and low community poverty level (AOR = 1.29, CI  =  1.07–1.56) had higher odds of using salt containing iodine.

**Conclusion:**

Both individual and community level factors were found to be associated with use of salt containing iodine in sub-Saharan Africa. Education level, media exposure, community poverty level, wealth index, community education, and community media exposure were found to be associated with use of salt containing iodine in SSA. Therefore, to improve the use of iodized salt in the region, there is a need to increase access to media sources and develop the socioeconomic status of the community.

## Introduction

Iodine is an essential trace mineral that is needed for the synthesis of thyroid hormones from the thyroid gland that aid in the development, growth and control of metabolic processes [1, 2]. When the intake of iodine is inadequate due to different reasons, the thyroid gland fails to synthesize sufficient amounts of thyroid hormones which in turn lead to the development of iodine deficiency disorders [3]. Iodine deficiency disorder (IDD) is associated with stillbirth, spontaneous abortion, congenital anomalies. perinatal mortality, cretinism, short stature, infant mortality, impaired mental functioning, delayed physical and psychomotor development [4–10]. It also affects quality of life the affected individual and economic productivity [11, 12].

Though the use of iodized salt can prevent iodine deficiency disorder, many parts of the world do not have enough iodine available through their diet and hence, iodine deficiency continues to be an important public health problem globally, making 30% of the world’s population at risk of iodine deficiency disorder [2]. This problem is more pronounced in sub-Saharan Africa [9]. To minimize the impact of IDD, the adoption of universal salt iodization (USI) program is recommend in all countries and there has been major global progress to the elimination of IDDs [13]. For instance, the global prevalence of clinical IDDs fell from 13.1% in 1993 to 3.2% in 2019, and 720 million cases of clinical IDDs have been prevented by USI (a reduction of 75.9%) [14] and the proportion of households consuming iodized salt increased from 70% in 2000 to 88% in 2019 low-income countries [13]. Although this substantial progress has been made over the last several decades towards iodized salt consumption, still it is under the recommendation of the World Health Organization(at least 90% of households should consume iodized salt) [8] and hence, iodine deficiency remains a significant health problem in developing countries [15].

Some previous research works conducted revealed that the use of iodized salt is associated with wealth index [16–19], age [16, 17], education status [16–20], community media exposure [18], knowledge of respondents on IDD and the importance of using iodized salt [20, 21]. Despite the use of iodized salt is a cost-effective universal strategy to combat IDD [22], IDD is still a problem in SSA [23]. However, to the best of our knowledge, there is no study that identifies the individual and community level determinants of iodized salt utilization in SSA. Identifying these factors that are associated with iodized salt utilization will be crucial for policy makers and other concerned bodies to take intervention measures and minimize the burden of IDD in the region.

## Methods

### Data source

The appended most recent demographic and health survey (DHS) datasets of 31 sub-Saharan countries (conducted from 2010 to 2018) were used for this study. The DHS is a nationally representative survey, collected every five years, to provide population and health indicators at the national and regional levels. A pretested standard Demographic and Health Survey questionnaires were used for data collection of the DHS surveys. The questionnaire was conceptualized to the different countries context and the data were collected by trained data collectors. The datasets of each sub Saharan country were obtained at https://dhsprogram.com/data/dataset_admin/index.cfm. Only those countries with data on iodized salt utilization were included for the analysis. Hence, a total weighted sample of 391,463 households were included in the analysis (Table 1).

**Table 1:** List of countries sub-Saharan countries, their demographic and health survey year and sample size included in the analysis.

Country name	Weighted sample size	Survey year
Angola	14,269	2015/16
Burkina	13,714	2010
Benin	13,288	2017/18
Burundi	14,389	2016/17
Central Democratic Congo	16,076	2013/14
Congo	10,453	2011/12
Cote d’vore	8,511	2011/12
Ethiopia	15,939	2016
Gabon	8,716	2012
Ghana	10,237	2014
Gambia	5,210	2013
Guinea	7,496	2018
Kenya	34,139	2014
Comoros	3,902	2012
Liberia	8,589	2013
Lesotho	6,583	2014
Mali	9,106	2018
Malawi	22,489	2015/16
Mozambique	13,140	2011
Nigeria	37,780	2018
Niger	9,010	2012
Namibia	9,205	2013
Rwanda	11,478	2014/15
Sera lone	11,322	2013
Chad	15,892	2014/15
Togo	8,958	2013
Tanzania	11,737	2015/16
Uganda	17,851	2016
South Africa	2,443	2016
Zambia	11,485	2018
Zimbabwe	8,056	2015

### Variables of the study

#### Dependent variable

Iodized salt utilization was the dependent variable. To ascertain the use of iodized salt, households were asked by the interviewers to bring a salt used for cooking purpose and they tested it using the iodine rapid test kits (RTKs) for presence or absence of iodine. So in this study, we only determined how much percentages of households were using salt that contains iodine irrespective of the amount of iodine content. And hence we dichotomized our dependent variable household iodized salt utilization into yes (households are using salt that has iodine) and no (households were not using salt that has iodine).

#### Independent variables

In this study, both individual and community level variables were included. The individual level factors included in this study were marital status, wealth index, education level, age of household head, and media exposure (a composite variable of having radio and television, in which households were said to have media exposure “if they have exposed to either of radio or television” and no “if did not have exposure to all of the above media sources”). The community level variables included in this study were residence, community poverty level (a composite variable generated from wealth index and defined as the proportion of households in the community with low wealth status (lowest and second quantiles), community education level and community media exposure(the proportion of households who had at least one of the medias sources (television or radio) and dichotomized as low(< 50% of households had one of these media sources) and high (≥ 50% of households had one of these media sources) based on median value)). With the exception of residence, the above mentioned community level variables were not directly found in the DHS data and hence they were created by aggregating their corresponding individual level variables at the cluster level. Then after, these generated community level variables were categorized as high and low based on their median value (their value were skewed).

*Data analysis procedure*. We used STATA 14 software to extract, recode and analyze the data. The data were weighted before doing any statistical analysis to restore the representativeness of the sample and get a reliable estimate and standard error. The procedure of weighting and its rationale is found on the guide of DHS statistics [24].

Due to the correlated nature of DHS data, measures of community variation/random-effects such as Intraclass Correlation Coefficient (ICC), Median Odds Ratio (MOR) and Proportional Change in Variance (PCV) were calculated. Accordingly, the values of these measures were found to be significant, and hence the use of multilevel logistic regression model is more appropriate than using ordinary logistic regression. To choose the best fitted model, first we developed four models and compared them with Deviance. These were: the null-model; a model with no independent variable, model I; a model that have individual-level factors only, model II; a model with community-level factors only and model III; a model that contain both individual and community level independent variables. Of the four models fitted, model III was the best fitted model, had the lowest Deviance.

After selecting the best fitted model, bivariable and multivariable multilevel logistic regression was done to determine the associated factors of iodized salt utilization in SSA. All variables with a p value < 0.25 at bi-variable analysis were entered into the multivariable multilevel logistic regression model. In the final model, we used p value ≤ 0.05 to declare statistically significant variables.

### Ethics consideration

We used a secondary analysis of DHS data. Therefore, obtaining ethical approval was not needed. However, we have received a permission letter to download and use the data files from DHS Program.

## Results

### Individual and community level characteristics of study participants

A total weighted samples of 391,463 households were included in this study. Of these, majority, 248,265 (63.42%) and 292,145 (74.63%) of them were living rural area and currently married, respectively. Regarding educational level, 133,352(34.06%) of the participants had not received formal education. Most of the study participants, 241,677(71. 74%) had media exposure. More than half of the study participants were from high community education (50.82%), and high community media exposure (51.14%) (Table 2).

**Table 2:** Socio demographic characteristics of respondents.

Variables	Weighted frequency	Percentage
Residence		
Urban	143,198	36.58
Rural	248,265	63.42
Marital status		
Never married	28,318	7.23
Married	292,145	74.63
Formerly married	71 000	18.14
Education level of household head		
No education	133,352	34.06
Primary education	125,493	32.06
Secondary education	100,546	25.68
Higher education	32,072	8.19
Community education		
Low	192,533	49.18
High	198,930	50.82
Wealth index		
Poorest	75,353	19.25
Poorer	76,352	19.50
Middle	77,364	19.76
Richer	80,028	20.44
Richest	82,366	21.04
Community poverty level		
Low	190,463	48.65
High	201,000	51.35
Sex of household head		
Male	284,605	72.70
Female	106,858	27.30
Age of household head (years)		
< 25	24,996	6.39
25–34	95,714	24.45
35–44	97,213	24.83
45–54	72,550	18.53
55–64	52,613	13.44
≥ 65	48,377	12.36
Media exposure		
Yes	241,677	61.74
No	149,786	38.26
Community media exposure		
Low	191,286	48.86
High	200,177	51.14

### Random effect analysis

According to the result of the random-effects model, there is significant clustering of iodized salt utilization across the communities (OR of community level variance = 2.75, 95% CI = 2.43–3.11). The value of ICC in the null model revealed that 45.49% of the overall variation of iodized salt utilization was attributed to cluster variability. The 4.83 MOR value of the null model also indicates the presence of variation in iodized salt utilization between clusters. It means if we randomly select households from different clusters, those households at the cluster with higher household iodized salt utilization had 4.83 times higher chance of using iodized salt use compared to their counterparts.

As you can see in the Table 3 below, the PCV increases from 12.64% (model I) to 31.97% (model III), indicating that model III best explains the variability of iodized salt utilization. Moreover, model III has the lowest Deviance value. Hence, it was selected as best fitted model (Table 3).

**Table 3:** Model comparison and random effect analysis result.

Parameters	Null model	Model I	Model II	Model III
Community level variance	2.75(2.43–3.11)	2.40(2.12–2.72)	2.02(1.79–2.28)	1.87(1.65–2.11)
ICC	45.49%	42.17%	38.00%	36.21%
MOR	4.83	4.36	3.85	3.66
PCV	Ref	12.64%	26.58%	31.97%
**Model fitness**				
Deviance	295231.02	291158.42	294384.88	290776.46

#### Associated factors of iodized salt utilization in sub-Saharan Africa

To identify associated factors of iodized salt utilization, both bivariable and multivariable multilevel logistic regression analyses were performed. Accordingly, on bivariable analysis, education level, marital status, media exposure, community media exposure, wealth index, community poverty and community education level were associated with iodized salt use in SSA (*p* < 0.25). In the final model, community poverty level, wealth index, community education, education level, and community media exposure were found to be significantly associated with iodized salt utilization (*p* ≤ 0.05).

Households who were from high community media exposure had 2.1 times higher odds of iodized salt utilization compared to those households of low community media exposure (AOR  =  2.07, 95% CI  =  1.71–2.51). Household heads with primary, secondary and higher education level had 1.5 (AOR  =  1.53, 95% CI  =  1.50–1.57), 1.8 (AOR  = 1.81, 95% CI  =  1.76–1.86) and 2.3 (AOR  =  2.28, 95% CI  =  2.17–2.40) times higher chance of using iodized salt compared to those with no education. Those households from high community education level had 3.8(AOR  =  3.78, 95% CI  =  3.14–4.56) times higher chance of iodized salt utilization compared to those households came from low community education level. Household with middle, richer and richest wealth status had 1.05 (AOR  =  1.05, 95% CI  =  1.02–1.08), 1.1 (AOR  = 1.13, 95% CI  =  1.09–1.17) and 1.2 (AOR  =  1.23, 95% CI  =  1.18–1.28) times higher odds of iodized salt utilization compared to households with poorest wealth index. Those households from low community poverty level had 1.3 (AOR = 1.29, CI  =  1.07–1.56) times higher odds of using salt containing iodine compared to their counterparts (Table 4).

**Table 4:** Multilevel logistic regression analysis of use of salt containing iodine in SSA, 2020.

Variables	Use of iodized salt		Odds ratio	
	Yes	No	COR	AOR
	N (%)	N (%)	(95% CI)	(95% CI)
Marital status				
Never married	25,500(90.05)	2,818(9.95)	1.26(1.21–1.31)	1.04(0.99–1.09)
Married	316,215(87.08)	46,930(12.92)	1.00	1.00
Media exposure				
Yes	214,095(88.59)	27,582(11.41)	1.21(1.19–1.23)	1.01(0.99–1.03)
No	127,619(85.20)	22,167(14,80)	1.00	1.00
Community media exposure				
Low				
High	165,212(86.37)	26,074(13.63)	1.00	1.00
	176,502(88.17)	23,674(11.83)	3.51(2.93–4.19)	2.07(1.71–2.51)*
Education level				
No education	110,452(82.83)	22,899(17.17)	1.00	1.00
1^ry^ education	110,596(88.13)	14,897(11.87)	1.56(1.53–1.60)	1.53(1.50–1.57)*
2^ry^ education	90,716(90.22)	9,830(9.78)	1.90(1.85–1.95)	1.81(1.76–1.86)*
Higher education	29,950(93.38)	2,122(6.62)	2.55(2.44–2.68)	2.28(2.17–2.40)*
Community education				
Low				
High	164,550(85.47)	27,983(14.53)	1.00	1.00
	177,165(89.06)	21,766(10.94)	5.45(4.55–6.54)	3.78(3.14–4.56)*
Wealth index				
Poorest	62,842(83.40)	12,511(16.60)	1.00	1.00
Poorer	64,932(85.04)	11,420(14.96)	1.03(1.01–1.06)	0.97(0.95–1.01)
Middle	67,376(87.09)	9,988(12.91)	1.15(1.12–1.19)	1.05(1.02–1.08)*
Richer	71,267(89.05)	8,762(10.95)	1.29(1.26–1.33)	1.13(1.09–1.17)*
Richest	75,299(91.42)	7,067(8.58)	1.60(1.55–1.65)	1.23(1.18–1.28)*
Community poverty				
Low	163,785(85.99)	26,678(14.01)	1.93(1.62–2.31)	1.29(1.07–1.56)*
High	177,930(88.52)	23,070(11.48)	1.00	1.00

*p≤ 0.05.

## Discussion

Though salt iodization is the preferred affordable strategy for prevention of iodine deficiency disorders [25], iodine deficiency disorders still continue to be a serious public health problem in low income countries [23, 26]. Therefore, this study was conducted to identify the associated factors of iodized salt utilization in SSA. This study found that education level, media exposure, residence, community poverty level, wealth index, community education, and community media exposure were associated with iodized salt utilization in SSA.

Similar to a study done in Ethiopia [18], in this study, those households from high community media exposure had an increased chance of using iodized salt compared to their counterparts. This is because media exposure is one effective way of increasing people’s level of awareness and use of iodized salt [27, 28]. This finding highlighted that the need to continual and effective use of media to improve the utilization of iodized salt in the region.

Another factor which is significantly associated with use of iodized salt is level of education. Those household with primary, secondary and higher education level had higher odds of using iodized salt compared to uneducated once. Similarly, households from high community education level had higher chance of using iodized salt compared to those households came from low community education level. The finding of this study is consistent with other studies conducted elsewhere [16–19]. This higher chance of using iodized salt among educated participants might be due an increased knowledge and awareness about the importance of using iodized salt [27, 29].

Consistent with previous studies conducted in low income countries [16–19, 30], in this study, wealth index is associated with use of iodized salt. Those households with middle, richer and richest wealth status had an increased chance of iodized salt utilization compared to households with poorest wealth status. Moreover, households who were from low community poverty level had higher odds of using iodized salt compared to their counterparts. This is because households’ access to use iodized salt is commonly dependent on awareness, availability and price [31]. Households with high economic status can afford to purchase salt from appropriate sources which contains iodine compared to poor households [32, 33].

This study has two major strengths. The first one is the use of very huge and representative datasets of 31 sub-Saharan countries, which are collected by well-trained data collectors using standard and validated questioner that makes the finding of this study to be generalizable for the region. The second strength of this study is the use of multilevel modeling, a model that accounts the nested/hierarchical nature the demographic and health survey to get reliable estimates. However, our study is not free from limitations. This study uses rapid test kits (RTKs) to identify weather the households are using salt that contains any levels of iodine or not. Therefore, we are unable to quantify the amount of iodine in the salt and hence, we failed to categorize the use of iodized salt as adequate or inadequate. Due to the secondary nature of the study, we are also unable to assess knowledge of individuals and some food sources of iodine with the use of iodized salt.

## Conclusion

Both individual and community level factors were found to be associated with use of salt containing iodine in sub-Saharan Africa. Education level, media exposure, community poverty level, wealth index, community education, and community media exposure were found to be associated with use of salt containing iodine in SSA. Therefore, to improve the use of iodized salt in the region, there is a need to increase access to media sources and develop the socioeconomic status of the community.

## Abbreviations

AOR: Adjusted Odds Ratio
CI: Confidence Interval
COR: Crude odds ratio
DHS: Demographic and Health Survey
ICC: Intraclass Correlation Coefficient
IDD: Iodine Deficiency Disorder
MOR: Median Odds Ratio
PCV: Proportional Change in Variance
SSA: Sub Saharan Africa
USI: Universal Salt Iodization
